# 

**Published:** 1880-02

**Authors:** 


Transactions of the American Medical Association. Vol. 30, 1879.

PAMPHLETS RECEIVED.—State Medicine and State Medical Societies. By Stanford E. Chaille, M.D., New Orleans, Louisiana.

Proceedings of the Louisiana State Medical Association at its second meeting, held in the city of New Orleans, April 9, 10 and 11, with constitution and by-laws.

